# Laser-Induced Graphene Electrodes for Wrist-Worn Impedance Plethysmography Measurements: A Feasibility Study

**DOI:** 10.3390/bios16080425

**Published:** 2026-08-06

**Authors:** Jorge A. Uc-Martín, Alejandro Cortés-Díaz-Sandi, Ilianny Castellón-Pérez, Roberto G. Ramírez-Chavarría

**Affiliations:** Instituto de Ingeniería, Universidad Nacional Autónoma de México, Mexico City 04510, Mexico; jorge.uc@comunidad.unam.mx (J.A.U.-M.); alexxander-2014@ciencias.unam.mx (A.C.-D.-S.); icastellonp@iingen.unam.mx (I.C.-P.)

**Keywords:** wrist-worn cardiac impedance sensor, laser-induced graphene, electrical impedance plethysmography (IPG), electrocardiogram, heart-rate estimation, non-invasive

## Abstract

Electrical bioimpedance (BioZ) has emerged as a promising technique for the non-invasive monitoring of physiological parameters, owing to its ability to map functional activity into electrical changes. Particularly, impedance plethysmography (IPG) is used to track blood volume changes associated with cardiac activity. However, developing flexible, low-cost devices with enough sensitivity to serve as high-precision for IPGs remains an open challenge. In this work, we introduce laser-induced graphene (LIG) electrodes as an attractive alternative for IPG measurements. The electrodes were fabricated by generating LIG on a polyimide substrate using a 405 nm laser diode and were subsequently characterized morphologically, structurally, and electrically to produce a wrist-worn cardiac impedance sensor (WCIS). The design of the WCIS is based on interdigitated electrodes to detect IPG variations at the radial artery, from which the heart rate is estimated. We show experimental results on IPG signal analysis and its validation against electrocardiogram (ECG) signals as the gold standard. As a result, a mean absolute error (MAE) of 1.7 bpm, a root mean square error (RMSE) of 2.1 bpm, and a limit of agreement of approximately ±6 bpm were obtained. These outcomes demonstrate the feasibility of the WCIS as a promising, low-cost alternative for continuous, non-invasive cardiovascular monitoring in portable devices, based on the IPG principle.

## 1. Introduction

Cardiovascular diseases (CVDs) encompass pathologies such as hypertension, heart failure, and coronary insufficiency, constituting a major public health challenge [1]. These conditions cause millions of deaths annually, according to reports from the European Society of Cardiology (ESC) and the World Health Organization (WHO) [2,3]. Consequently, continuous monitoring of cardiovascular and respiratory parameters enables early diagnosis of CVDs, facilitating timely medical interventions. This continuous assessment also optimizes therapeutic decision-making in critically ill patients [4]. In this context, point-of-care (PoC) diagnostics are a fundamental requirement, enabling continuous, non-invasive monitoring [5,6,7]. The above enhances real-time patient evaluation, providing essential indicators to both users and clinicians for low-cost early healthcare (e-Health) [8].

Electrocardiography (ECG) constitutes the traditional method for evaluating the electrical activity of the heart [9,10]. This technique represents a rapid, non-invasive, and highly convenient tool in hospital environments. Its analysis enables the detection of alterations in the PR interval, the QRS complex, the ST segment, the T-wave amplitude, and the corrected QT interval, which are fundamental parameters for CVD diagnosis. However, these signals may be difficult to recognize, even for experienced cardiologists, in patients presenting perceptible symptoms [11]. Other non-invasive techniques include sphygmomanometry, photoplethysmography, and Doppler echocardiography. Nonetheless, these methodologies present distinct limitations, such as motion artifacts and inter-operator variability, which require periodic calibration [12,13,14].

Commercial wearable health monitoring technologies have become widespread in recent years. Devices such as the Apple Watch, Fitbit and Garmin measure heart rate, perform electrocardiograms and estimate blood oxygen saturation, among other physiological signals, mainly through photoplethysmography (PPG) [15,16]. Although this technique is well validated at rest, its accuracy degrades during physical activity due to motion artifacts [17,18], and its dependence on an active light source increases energy consumption, limiting battery life in continuous monitoring [19]. Furthermore, the influence of skin tone on its accuracy remains a matter of debate [20,21], and its implementation requires comparatively complex architectures by integrating optical hardware with dedicated signal processing software [22].

In this regard, the development of wearable health monitoring systems (WHMSs) offers new perspectives and robust methodologies for non-invasive cardiac monitoring, providing easily interpretable biosignals [23]. Consequently, WHMS research is focused on exploring new, low-cost materials and technologies for daily biosignal recording.

In this search for new materials, various conductive materials have been explored for the fabrication of flexible electrophysiological monitoring electrodes, each defined by both its intrinsic properties and its associated fabrication method. Silver nanowire (AgNW) networks offer high conductivity and optical transparency [24,25], but exhibit high contact resistance at the junctions and poor long-term stability, requiring additional welding and protective coating steps [24]. Liquid metal (LM) electrodes are notable for their softness, elasticity, and high conductivity, enabling high-resolution cardiac electrogram mapping [26]; however, their high-resolution patterning requires multi-step processes and encapsulation to prevent leakage and oxidation [26]. Other alternatives include carbon nanotubes (CNTs) [27,28] and reduced graphene oxide (rGO) [29,30].

In contrast, laser-induced graphene (LIG) is obtained in a single step, without a mask, by direct laser irradiation of polymeric substrates, without chemical reagents or cleanroom infrastructure [31,32]. This confers on LIG several key advantages for electrophysiological applications: (i) low cost and short fabrication time, reducing the cost by more than two orders of magnitude compared to conventional neural electrode fabrication [31]; (ii) scalability to mass production without complex equipment or templates [31,33]; (iii) digital customization of electrode geometry directly from a file, allowing specific patterns such as fractal ECG electrodes [32]; and (iv) intrinsic chemical stability against corrosion in humid environments [33], unlike oxidation-prone AgNW junctions [24] or leak-prone LM interfaces [26].

Additionally, LIG-based transducers prominently feature in the development of low-power WHMS due to their excellent electrical conductivity ($5–25\;{\mathrm{Scm}}^{-1}$), mechanical flexibility, and high active surface area (∼$340\;m^{2}\;g^{-1}$) [34,35]. These characteristics enhance transducer sensitivity and charge transfer in biosensing applications. Another advantage over conventional alternatives resides in a straightforward, reagent-free fabrication process for entirely flexible structures. This technical versatility underscores the potential of LIG to adapt and maintain prolonged skin contact across complex anatomical regions [1].

However, the final properties of the LIG depend critically on the technique used to generate it, particularly the wavelength of the laser employed. Conventional LIG generation techniques encompass ultraviolet (UV), visible, and infrared (IR) laser systems. Among these, IR systems operating at a wavelength of λ=10.6μm represent the most widely utilized method [36]. Due to its long wavelength, the technique relies predominantly on photothermal conversion, requiring a critical fluence of approximately $4.9\;{J/\mathrm{cm}}^{2}$ to initiate carbonization and thereby induce heat-affected zones that can ablate thermally sensitive substrates. Furthermore, the Gaussian energy distribution within the laser beam causes the focal center to reach higher temperatures than the margins, resulting in edges with a higher defect density and reduced electrical conductivity compared to the engraved center [37]. Consequently, using lasers with λ=405nm emerges as a prominent alternative to the traditional method, gaining popularity for its low cost, compact size, extended operational lifespan, and high energy efficiency. At this wavelength, photochemical reactions directly cleave chemical bonds, enabling highly precise carbonization [38]. Nevertheless, the literature highlights the need for further exploration regarding the integration of 405nm LIG into sensor development and related applications [38].

Based on the above rationale, this work introduces a wrist-worn cardiac impedance sensor (WCIS) fabricated from LIG at λ=405nm, along with a methodology for wrist-based heart rate estimation. This sensor leverages the high electrical conductivity and straightforward fabrication of LIG, demonstrating that the 405nm wavelength enables the development of sensors optimized for cardiovascular parameter monitoring [39]. The WCIS features an interdigitated electrode (IDE) structure coupled to a MAX3000x device (Analog Devices, Inc., Wilmington, NC, USA) that applies an excitation signal and measures variations in electrical bioimpedance (ΔBioZ). From these recorded variations, impedance plethysmography (IPG) data are extracted to quantify transient changes in blood volume throughout the cardiac cycle, and the results are validated against electrocardiography (ECG).

In this context, the WCIS measures pulsatile hemodynamic variations in the radial artery using a localized electric field detection method, generating an electrical signal from which the heart rate is extracted after signal conditioning and filtering. This approach is related to wearable bioimpedance and fringing-field capacitive sensing techniques for non-invasive cardiovascular monitoring [40,41,42]. These techniques have been reported to offer a simpler sensing architecture, with the potential for lower manufacturing cost and high sensitivity, compared to the optical architectures used in commercial PPG-based systems [43].

Unlike conventional multi-electrode systems that require conductive gels, the proposed methodology employs a single IDE structure positioned non-invasively on the anterior aspect of the wrist, offering a portable, ergonomic design that facilitates a transition toward decentralized, personalized healthcare, and is presented as a complementary and reproducible approach relative to PPG-based commercial devices, offering two potential advantages: (i) a simpler architecture requiring less specialized hardware/software integration than PPG systems; and (ii) a low-cost, scalable graphene-based fabrication process. Additionally, since the sensor operates through a passive mechanical-to-electrical transduction mechanism, without an active optical emitter, it may in principle offer reduced power consumption relative to actively-illuminated PPG systems; however, this potential benefit has not yet been quantified in our system and is left as a direction for future work.

The contributions of this article are as follows:

1. The fabrication of laser-induced graphene (LIG) for bioimpedance applications using a 405nm laser, whose use for LIG fabrication has been previously reported [44,45], yielding a homogeneous chemical and structural composition validated by Raman spectroscopy. The resulting D, G, and 2D peaks exhibit errors below 0.32% relative to values reported for 450nm laser-generated LIG.
2. The design and fabrication of a LIG-based wrist-worn cardiac impedance sensor that successfully transduces pulsatile hemodynamic activity into a quantifiable signal, relying on a single electrode structure to offer a more practical alternative to conventional three-electrode configurations.
3. A non-invasive cardiac measurement methodology that presents a limit of agreement (LoA) of approximately 6bpm and a signal-to-noise ratio (SNR) of 83.4dB, positioning the device within the performance specifications required for precision physiological monitoring.

This article proceeds as follows. Section 2 details the materials, the electrode manufacturing process, and the experimental methodology. Section 3 presents and discusses the results. Section 4 summarizes the main conclusions.

## 2. Materials and Methods

### 2.1. Electrode Design and Fabrication

**Figure 1:**
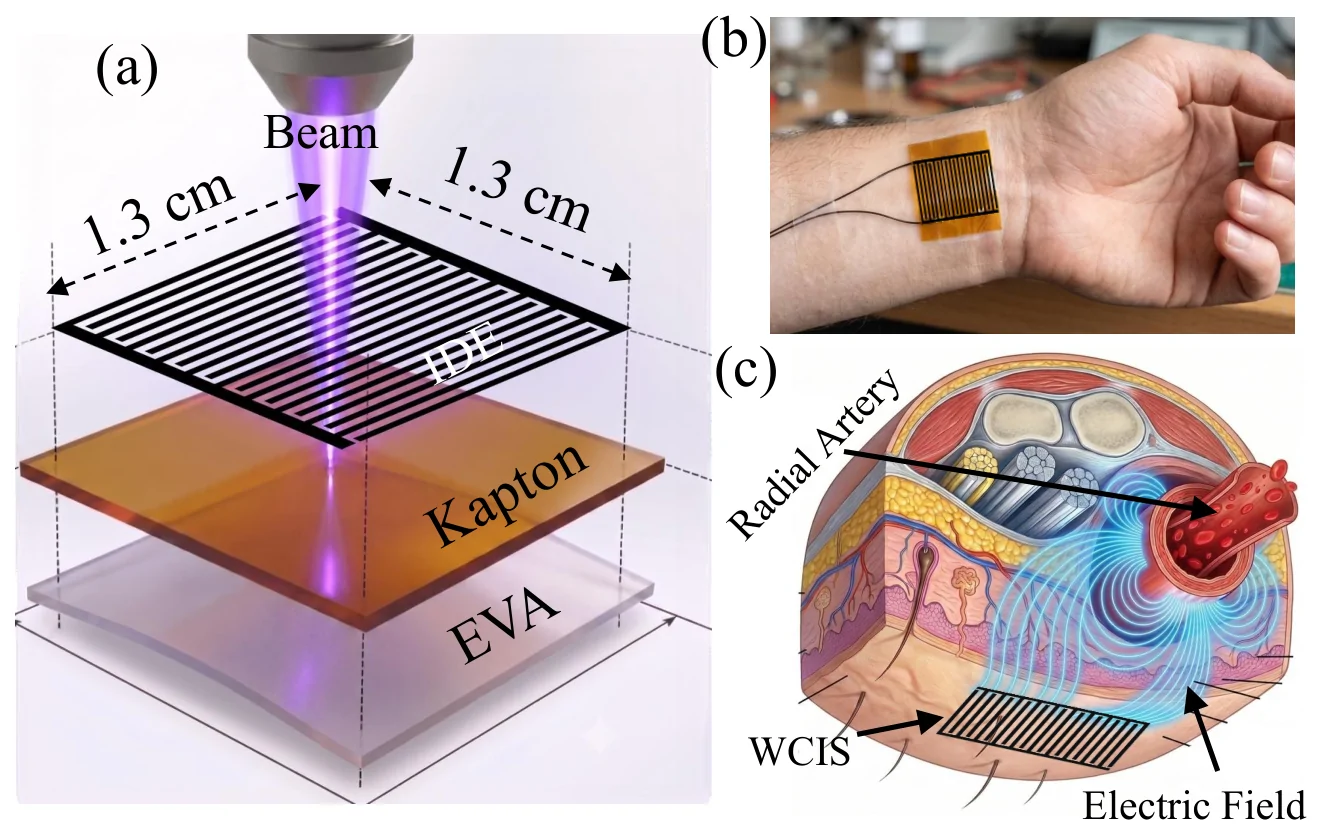
Wrist-worn cardiac impedance sensor. (**a**) Manufacturing process. (**b**) Position on the wrist. (**c**) Interaction of the electric field with the radial artery.

The WCIS is fabricated utilizing LIG technology on a commercial polyimide (PI), namely Kapton, substrate with a total thickness of 60μm, where a λ=405nm laser beam induces the localized conversion of the polymer surface into a conductive graphene structure. Prior to the LIG synthesis process, the PI film is attached to an ethylene-vinyl acetate (EVA) sheet, as illustrated in Figure 1a. The sensor features an interdigitated electrode (IDE) geometry with a sensing area of approximately $1.7\;{\mathrm{mm}}^{2}$ and a finger spacing of 1mm. This geometric arrangement provides a larger effective contact area. It promotes a more uniform distribution of the electric field ($\vec{E}$) across the entire sensing surface, thereby facilitating penetration through different skin layers to reach the underlying tissue of interest [46]. Additionally, the design includes two small rectangular pads that facilitate cold soldering using a transparent commercial epoxy adhesive (Pegatanke, Manta, Ecuador) to connect wires to the measurement system.

As depicted in Figure 1b, the sensor is positioned comfortably on the wrist, eliminating the need for conductive gels between the electrodes and the skin. Finally, Figure 1c provides a schematic representation of the generated $\vec{E}$ interacting with the anatomical medium, explicitly illustrating its path through the distinct skin layers into the target underlying tissue.

### 2.2. Measurement Principle

The physiological principles enabling the measurement of hemodynamic signals with the WCIS rely on localized tissue–field interactions. The sensor operates by detecting the interaction between the electric field generated by the interdigitated electrodes and the pulsatile variations in arterial blood volume that occur with each heartbeat. During systole, the heart contracts, propelling pulsatile blood flow through the arterial network, including the radial artery, which runs along the anterior surface of the wrist to carry oxygenated blood from the aorta to the hand. During each cardiac cycle, a temporary increase in blood flow slightly dilates the arterial diameter, providing a physiological variation leveraged by the WCIS when positioned on the ventral aspect of the wrist directly over the radial artery, as illustrated in Figure 1c.

Now, exposing the sensor to air and applying a potential difference ($V_{0}$) between two electrode fingers separated by a distance (*d*) generates a uniform electric field (${\vec{E}}_{0}$) on its sensing surface, allowing its description under a quasi-static approximation as E→0=V0d. When the WCIS is placed on the wrist, the electric field no longer interacts solely with air but extends into the underlying biological tissues. In particular, the blood circulating through the radial artery modifies the effective electrical properties of the medium detected by the device, characterizing the sensing domain through its electrical conductivity ($\sigma_{\mathrm{blood}}$) and dielectric permittivity ($\epsilon_{\mathrm{blood}}$). With each heartbeat, the blood volume within the radial artery undergoes cyclical variations, producing transient changes in the effective electrical properties of the medium ($\Delta \epsilon_{eff}$, $\Delta \sigma_{eff}$). These variations alter the distribution of the electric field generated by the sensor, resulting in a total dynamic field $\vec{E}\left(t\right)$ expressed as $\vec{E}\left(t\right)={\vec{E}}_{0}+\Delta \vec{E}\left(t\right)$, where $\Delta \vec{E}\left(t\right)$ represents the disturbance induced by the arterial pulse. Consequently, these volumetric and dielectric fluctuations translate into a measurable dynamic impedance variation (ΔZ), governed by the following volume integral [47,48]

(1) $$\Delta Z=-\frac{1}{I^{2}}\int_{V}\Delta \gamma_{eff}\left(t\right)\left({\vec{E}}_{0}\;\cdot \;\vec{E}\left(t\right)\right)dV$$

where *I* represents the excitation current, ${\vec{E}}_{0}$ denotes the baseline static electric field, and $\Delta \gamma_{eff}\left(t\right)$ corresponds to the transient variation in the effective complex admittance of the tissue ($\Delta \sigma_{eff}+j\omega \Delta \epsilon_{eff}$).

Therefore, the IDE acts as a transducer that converts pulse-induced variations in the electrical properties of the tissue into measurable impedance fluctuations. These impedance changes are acquired by the electronic interface and represented as a time-dependent waveform that reflects hemodynamic activity.

### 2.3. Instrumentation and Signal Acquisition

**Figure 2:**
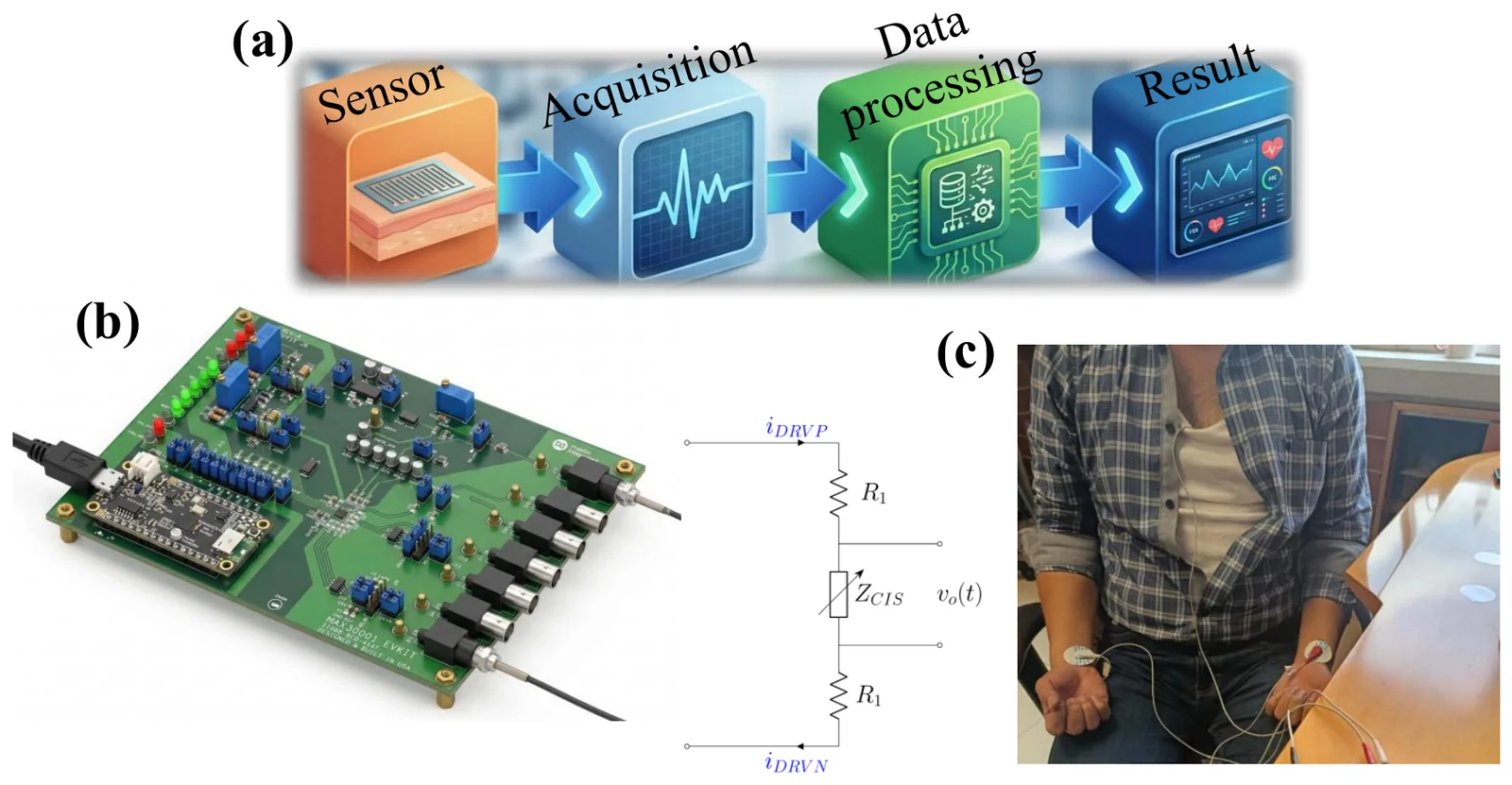
Proposed methodology for heart-rate estimation. (**a**) Block diagram. (**b**) System configuration. (**c**) Traditional method for evaluating cardiac electrical activity (ECG).

Figure 2a shows the proposed measurement methodology, comprising four stages that enable continuous, non-invasive cardiovascular monitoring using the WCIS [43]. The sensing stage involves positioning the WCIS on the wrist, establishing a fixed skin contact, and aligning it directly over the radial artery. Subsequently, the MAX30001 evaluation system (Analog Devices) is responsible for signal acquisition, enabling real-time visualization and data recording for further processing. The third stage, data processing, implements signal filtering for noise and artifact removal, and peak detection to accurately locate systolic events. Finally, the output provides an estimated heart rate in beats per minute (bpm), a clinically relevant metric.

Figure 2b displays the MAX30001 evaluation system (Analog Devices) [49], which consists of an ultra-low-power analog front-end (AFE) integrated circuit designed for healthcare applications. This AFE incorporates two channels, enabling parallel, synchronized recording of BioZ and ECG signals.

The bioimpedance channel operates via an external voltage-divider circuit connected to the board, injecting a square-wave current excitation signal ($i_{DRVP}$) through resistor $R_{1}$ into the wrist-worn sensor ($Z_{CIS}$). The output voltage $v_{o}\left(t\right)$ defines the sensor response, directly reflecting the electrical changes induced by transient physiological fluctuations in blood volume along the radial artery. For this purpose, the output voltage $v_{o}\left(t\right)$ is conditioned and processed as follows. An analog high-pass filter (HPF), with a cutoff frequency of 1kHz, attenuates low-frequency noise and direct current (DC) offsets at the electrodes. Meanwhile, an instrumentation amplifier (INA) is configured in low-noise mode to reduce thermal noise. At the same time, the programmable gain amplifier (PGA) is configured with a channel gain of 10V/V to match the dynamic range of a 20-bit analog-to-digital converter (ADC). A sampling rate of 32samples/s defines a stable data output. Additionally, the digital low-pass filter (DLPF) (4Hz) and DHPF (0.5Hz) constrain the bandwidth for physiological event detection, eliminating out-of-band interference. For tissue excitation, the system injects a current of 96μA at a frequency of 128kHz. Finally, the current generator mode, configured as chopped w/o LPF, is recommended for switching to prevent impedance measurement distortions, with the current monitor disabled and the internal resistor reference used for generator biasing.

Further, a MAX32630FTHR microcontroller (ARM Cortex-M4F architecture) manages the entire channel data flow via serial peripheral interface (SPI), thus allowing the MAX30001 module to translate the acquired signals into impedance values according to the following relationship:

(2) $$\Delta Z\left(t\right)=ADC_{\mathrm{value}}\;\cdot \;\frac{V_{REF}}{2^{19}\;\cdot \;CG_{MAG}\;\cdot \;Gain}$$

where $ADC_{\mathrm{value}}$ is the raw data provided by the internal analog-to-digital converter (ADC), $V_{REF}$ is the internal reference voltage, $CG_{MAG}$ is the magnitude of the selected excitation current, Gain is the selected gain for the BioZ channel, and $2^{19}$ is the scaling factor of the 20-bit ADC. Table 1 summarizes the measurement configuration for the MAX30001 bioimpedance (BioZ) channel.

For comparison purposes, the ECG channel was also used and configured according to the parameters in Table 2. Here, the 20V/V channel gain amplifies the signal, matching it to the ADC’s dynamic range. On the other hand, the sampling rate (512samples/s) ensures high temporal resolution for precise QRS complex identification. The digital LPF cutoff frequency is set to 40.96Hz, whereas the DHPF cutoff equals 0.5Hz for eliminating high-frequency interference and baseline wander caused by respiration or user movement, respectively. Finally, the normal fast recovery mode operates alongside its threshold, i.e., 63×2048LSB, stabilizing the biosignal following the detection of large-amplitude transients at the electrode interface.

## 3. Results and Discussion

### 3.1. Evaluation of the Electrode Manufacturing Process

**Figure 3:**
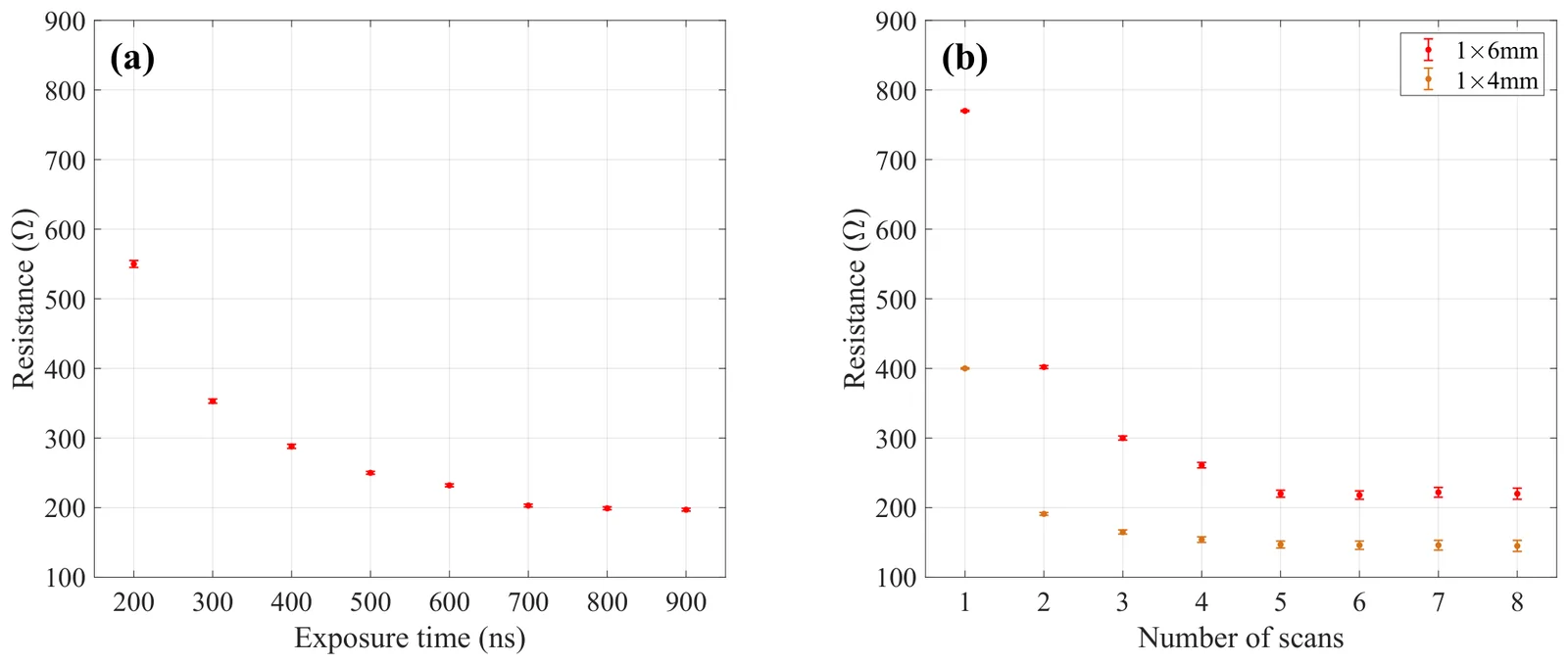
Effect of manufacturing parameters on WCIS resistance. (**a**) Resistance as a function of laser exposure time. (**b**) Resistance as a function of the number of scans.

Ten WCISs were fabricated using LIG with identical geometric patterns, with laser exposure time and the number of scanning cycles varied to evaluate the intrinsic resistance of each sensor, $R_{CIS}$. The measured resistance values as a function of these fabrication parameters are shown in Figure 3, which illustrates the relationship between the laser processing conditions and the resulting sensor resistance.

Figure 3a shows that the WCIS resistance decreases as the laser exposure time increases, a trend similarly observed as a function of the number of scanning cycles. Beyond 700 ns, the resistance stabilizes at approximately 200 Ω, indicating that the LIG formation process has reached a saturation regime, in which additional exposure time no longer contributes significantly to structural transformations or the generation of new conductive pathways within the material. In turn, longer exposure times caused partial detachment of the LIG from the PI surface, compromising the correct synthesis. For this reason, the exposure time was set to the system default of 500 ns.

Prior to fabricating the final WCIS pattern, the fabrication parameters were characterized using rectangular LIG test structures. To verify that the decreasing and saturation trend of $R_{CIS}$ observed as a function of the number of scanning cycles was independent of the pattern geometry, two of these structures with different dimensions (1 × 4 mm and 1 × 6 mm) were evaluated, as shown in Figure 3b, where the first value corresponds to the fixed width and the second to the rectangle length. In both cases, $R_{CIS}$ exhibited the same decreasing trend followed by saturation, although the 1 × 6 mm sample consistently showed higher resistance values than the 1 × 4 mm sample, consistent with the linear dependence of resistance on length (R∝L) for a constant cross-sectional area. These results confirm that the pattern length determines the absolute value of $R_{CIS}$, whereas the saturation observed after the fifth scan is an intrinsic feature of the LIG formation process, independent of the sample dimensions.

Specifically, Figure 3b shows that $R_{CIS}$ exhibits an approximately exponential decrease with the number of scanning cycles. In a single scan, the resistance reaches approximately 800 Ω, dropping by nearly half after the second cycle—the stage at which the most significant improvement in material conductivity occurs, attributed to increased LIG formation that occurs in areas left unprocessed during the first scan. From scan 3 to scan 5, the rate of decrease in $R_{CIS}$ becomes progressively less pronounced, suggesting that prior scans had already activated the majority of available regions for LIG formation. Beyond five scans, the resistance stabilizes, indicating saturation of the material formation process. Based on these results, 5 scanning cycles were selected as the optimal manufacturing parameter. This represents an optimal trade-off between low electrical resistance and preservation of pattern resolution, while ensuring reproducible $R_{CIS}$ values under equivalent geometric and fabrication conditions.

### 3.2. Structural Analysis

**Figure 4:**
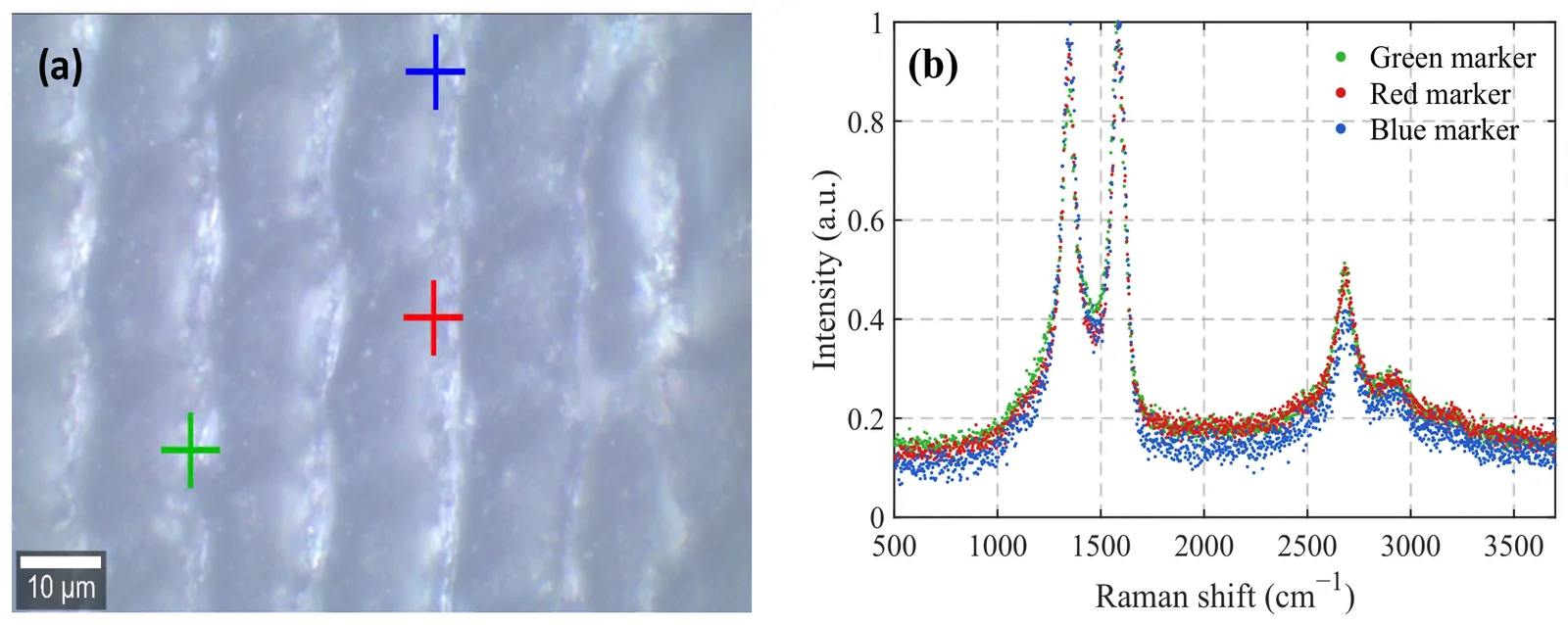
Raman analysis of the LIG electrodes. (**a**) Laser-induced graphene points selected for Raman spectra. (**b**) Raman spectra of the three points.

Once the WCIS were fabricated, the structural analysis was conducted using Raman spectroscopy. The Raman spectra were acquired using a 532 nm excitation laser, with a laser power of 3.56 mW, an integration time of 0.3 s, 30 accumulations, and a 100× objective lens. This task was performed on three different areas of the LIG-based electrodes. The results obtained from the Raman analysis are shown in Figure 4b.

Figure 4a shows an optical image of the LIG surface obtained by microscopy, indicating three representative measurement points marked in blue, red, and green. The 10 μm scale highlights the material’s characteristic fibrous structures at the microscopic scale. The Raman spectra acquired at each of these points are presented in Figure 4b, which shows three local maxima that, from left to right, correspond to the D (∼1350 ${\mathrm{cm}}^{-1}$), G (∼1580 ${\mathrm{cm}}^{-1}$), and 2D (∼2700 ${\mathrm{cm}}^{-1}$) bands, characteristic of graphene and derived materials. The remarkable coincidence in the peak positions among the three measurement points indicates a homogeneous chemical and structural composition across the LIG surface. Furthermore, the location of these peaks is independent of the number of scans and the exposure time used during fabrication, suggesting that these parameters do not alter the material’s crystalline structure. Table 3 compares the Raman shift values obtained with those reported in the literature for LIG fabricated with a 450 nm laser [50].

As shown in Table 3, the Raman shift values obtained for the D, G, and 2D peaks closely agree with those reported in the literature for LIG fabricated with a 450 nm laser [50], with relative errors of 0.06%, 0.21%, and 0.32%, respectively. These small deviations confirm that the spectral characteristics are consistent with those of LIG, despite the difference in laser wavelength. Similar Raman peak positions have also been reported for LIG fabricated using other visible-wavelength lasers. For example, LIG produced with a 532 nm laser exhibited D, G, and 2D peaks at 1341, 1579, and 2682 ${\mathrm{cm}}^{-1}$, respectively, [51], while LIG fabricated using a 450 nm diode laser showed peaks at 1330, 1590, and 2660 ${\mathrm{cm}}^{-1}$ [52]. Although slight variations in peak position are observed among these studies, they are expected due to differences in laser wavelength, irradiation conditions, precursor materials, and the degree of graphitization. Furthermore, the relative peak intensities follow the same trend commonly reported for LIG [50,51,52]: the D and G bands exhibit comparable intensities, both significantly higher than that of the 2D band, which is characteristic of defective or few-layer graphene structures.

### 3.3. Morphological Analysis

**Figure 5:**
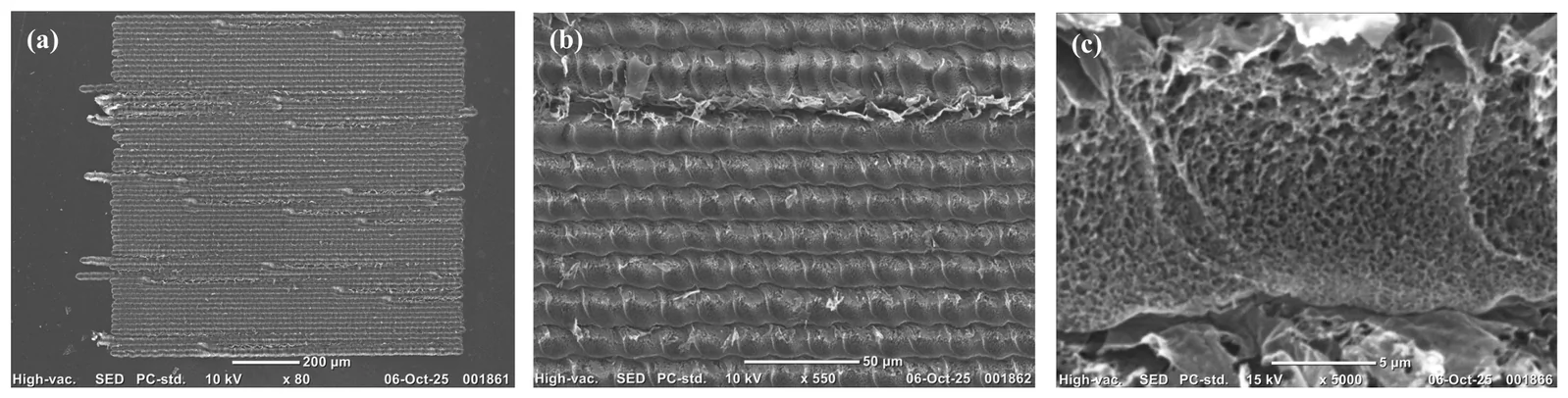
SEM images of the LIG at different magnifications. (**a**) 80× magnification, (**b**) 550× magnification, (**c**) 5000× magnification.

To go further, the electrode surface was morphologically studied. For this purpose, a JEOL JSM-IT500 field-emission scanning electron microscope (FESEM) (JEOL USA, Inc., Peabody, MA, USA) at 10,000× magnification was used. Figure 5 shows the images obtained using FESEM.

In Figure 5a, horizontal patterns of LIG can be observed, corresponding to the way the laser moves to synthesize the LIG. These horizontal patterns can be seen in greater detail in Figure 5b. On the other hand, in Figure 5c, the characteristic pores of the LIG are visible. Using ImageJ software (version 1.54t), the average diameter of the graphene pores was determined to be (1.02±0.17)μm. The literature reports that LIG fabricated with a ${\mathrm{CO}}_{2}$ laser has a pore diameter of 4–8 nm. On the other hand, for the case of LIG fabricated with a 355 nm UV laser, nanometric pores are obtained [53]. Therefore, considering that the 405 nm wavelength of the laser used in this work is less than that of the ${\mathrm{CO}}_{2}$ laser but greater than that of a UV laser, it is consistent that the pore diameter obtained is also located within the range reported for each laser.

Comparing the results obtained from the morphological and structural characterization with those reported in the literature, it is inferred that the material produced through the irradiation of a polymer with a 405 nm laser diode exhibits properties consistent with LIG.

### 3.4. Electrical Characterization

**Figure 6:**
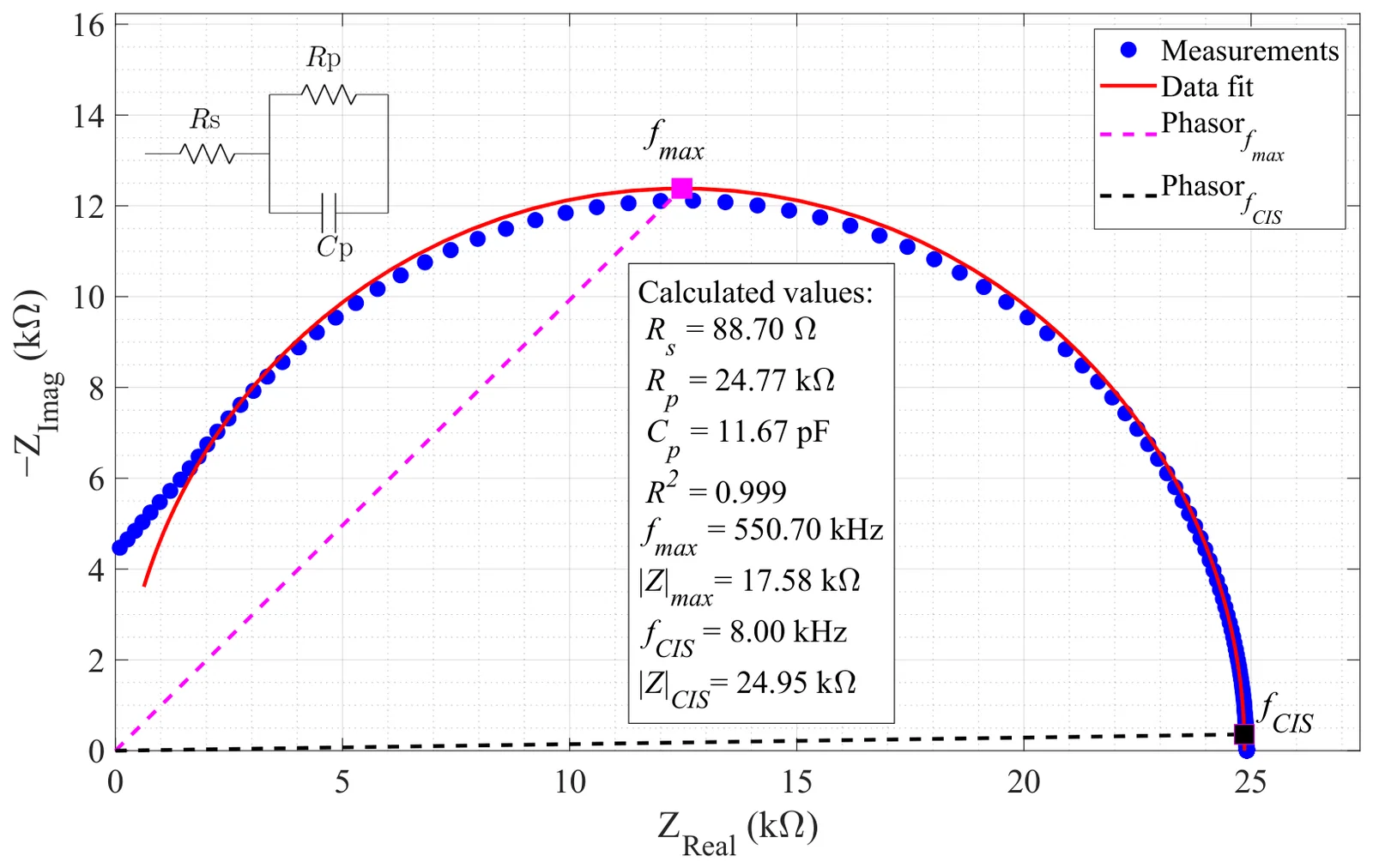
Nyquist diagram of the WCIS. Magenta and black dashed lines: impedance vectors for the condition $f_{max}$ and the operating point at $f_{CIS}$ kHz, respectively.

Once the material’s structural and morphological characteristics have been confirmed, it is necessary to evaluate its electrical properties, as these determine its performance as a sensing element. In particular, the analysis of the frequency response enables the identification of charge-transport mechanisms and the modeling of the equivalent electrical behavior. For this purpose, electrical impedance spectroscopy (EIS) [54] was used to characterize the sensor’s electrical behavior over the frequency range of 100 Hz to 5 MHz using a ZM2376 LCR meter (NF Corporation, Yokohama, Japan). The measurement was conducted by exposing the sensor to air in the absence of electrolyte, under stable yet uncontrolled environmental conditions (22 °C, 40% HR). The sensor was connected to the LCR meter using a four-terminal connection topology (Generic 4TP test device) with an applied voltage of 1 Vrms. Therefore, EIS allows the complex impedance of the sensor to be obtained as ZCIS=Re{ZCIS}+jIm{ZCIS}. The real component, Re, was then plotted against the imaginary component, Im, generating the Nyquist diagram shown in Figure 6 where blue markers correspond to the experimental data and the red curve represents the fit obtained from an equivalent circuit model (ECM), as shown in the inset of Figure 6.

As a result, the equivalent circuit parameters were $R_{s}$ = 88.703 Ω, $R_{p}$ = 24.767 kΩ, and $C_{p}$ = 11.6 pF. The low value of $R_{s}$ indicates a small contribution from ohmic losses. This characteristic benefits signal transmission and minimizes energy dissipation in the device. On the other hand, the high value of $R_{p}$ compared to $R_{s}$ suggests a low leakage current through the material. This condition allows the sensor’s capacitive behavior to predominate within the studied frequency range. Furthermore, the obtained capacitance reflects the device’s ability to store electrical charge at the electrode interface. This feature is fundamental for its operation as an impedimentric sensor. Altogether, these results confirm that the ECM structure is correctly modeling the electrical response of the WCIS. Therefore, this outcome demonstrates that charge–storage processes, rather than purely resistive mechanisms, dominate the electrical response of the electrodes.

To go further, using the Nyquist diagram in Figure 6, it was possible to determine the frequency at which WCIS must operate according to the MAX30001 specifications. For this purpose, the impedance phasor was determined at $f_{CIS}=8\;\mathrm{kHz}$, where the Nyquist diagram yields Re{ZCIS}=24.897kΩ and Im{ZCIS}=397.652Ω (see the black straight line in Figure 6). Interestingly, this value corresponds to a characteristic frequency of. Because the imaginary component accounts for less than 2% of the real component, the latter dominates the total impedance magnitude, thereby establishing a predominantly resistive behavior.

### 3.5. Bioimpedance Measurement Analysis

Given the previous analysis, we know from EIS that the WCIS has an approximate value of ${\left|Z\right|}_{CIS}=24.9$ kΩ. With this at hand, the WCIS signal was acquired online using the MAX30001 analog front-end integrated circuit, operating at a sampling frequency of $f_{s}=32$ Hz and $f_{CIS}=8\;\mathrm{kHz}$. The purpose of this experiment was to validate the sensor’s accuracy and noise distribution under static, well-known conditions.

**Figure 7:**
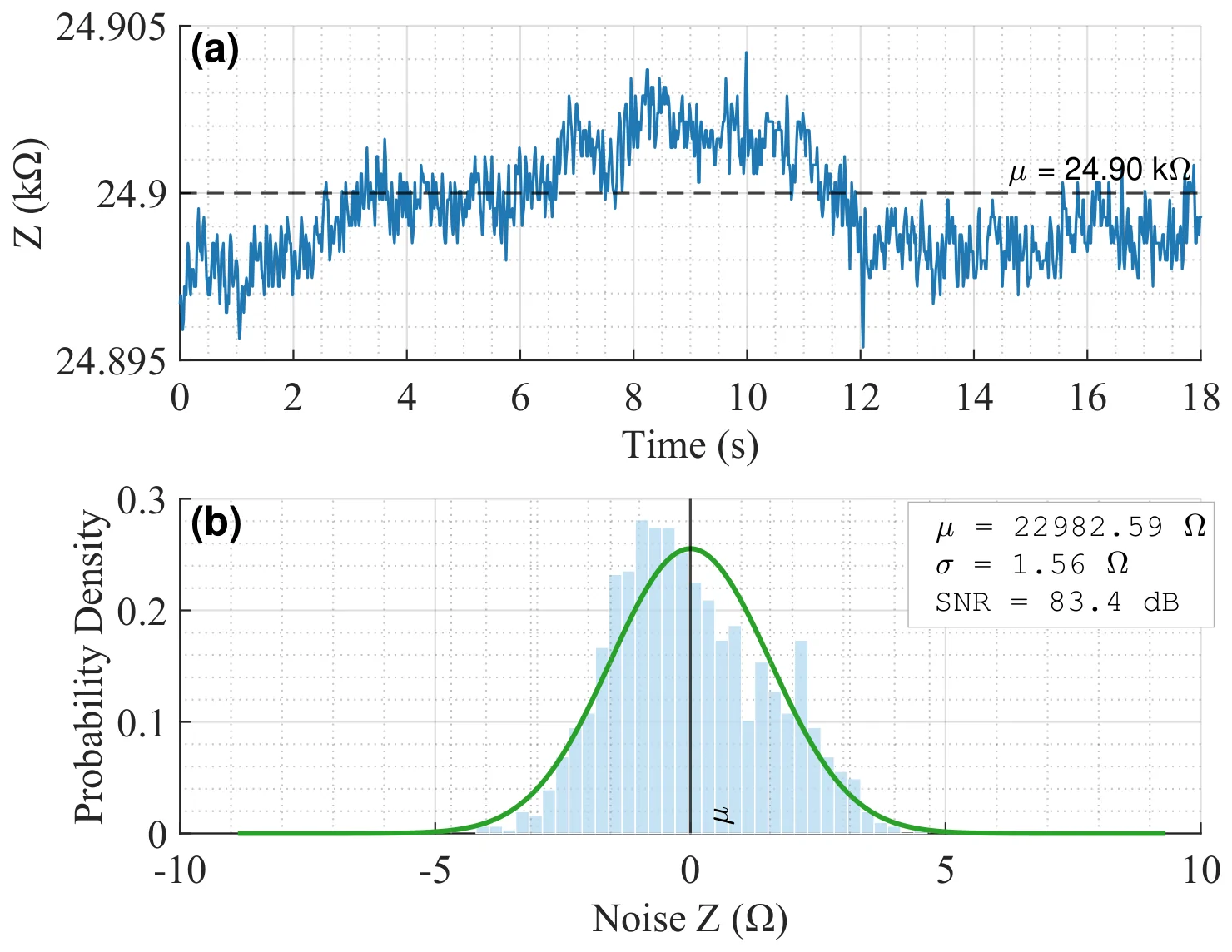
Bioimpedance data acquisition and signal analysis. (**a**) Raw signal acquired with the MAX30001. (**b**) Statistical analysis of the residual noise component.

Figure 7a displays the temporal evolution of the measured bioimpedance signal over a window of approximately 18 s. The horizontal dotted line marks the true impedance value computed from the previous EIS experiment. The signal measured by the MAX30001 remains within an extremely narrow amplitude range, which confirms the high measurement precision of the integrated circuit. The annotated standard deviation (σ=1.56Ω) corresponds to a relative variability of approximately $6.8\times 10^{-3}$%, which underscores the sensor’s stability in the absence of physiological modulation. Figure 7b shows the statistical distribution of the residual noise component ΔZ. The horizontal axis spans a noise range from approximately −10 to +10Ω, while the vertical axis represents the probability density. The empirical histogram is overlaid with a nearly Gaussian fitted probability density function, confirming that the noise is predominantly stochastic and well-behaved. The distribution centers at μ≈0Ω, with a noise standard deviation (σ=1.56Ω) characterizing the dispersion. The panel directly reports the signal-to-noise ratio as SNR=83.4dB, reflecting the high fidelity of the MAX30001 BioZ acquisition channel. This figure of merit places the system well within the performance specifications required for precision bioimpedance-based physiological monitoring applications.

### 3.6. Wrist-Based IPG Signal Analysis

**Figure 8:**
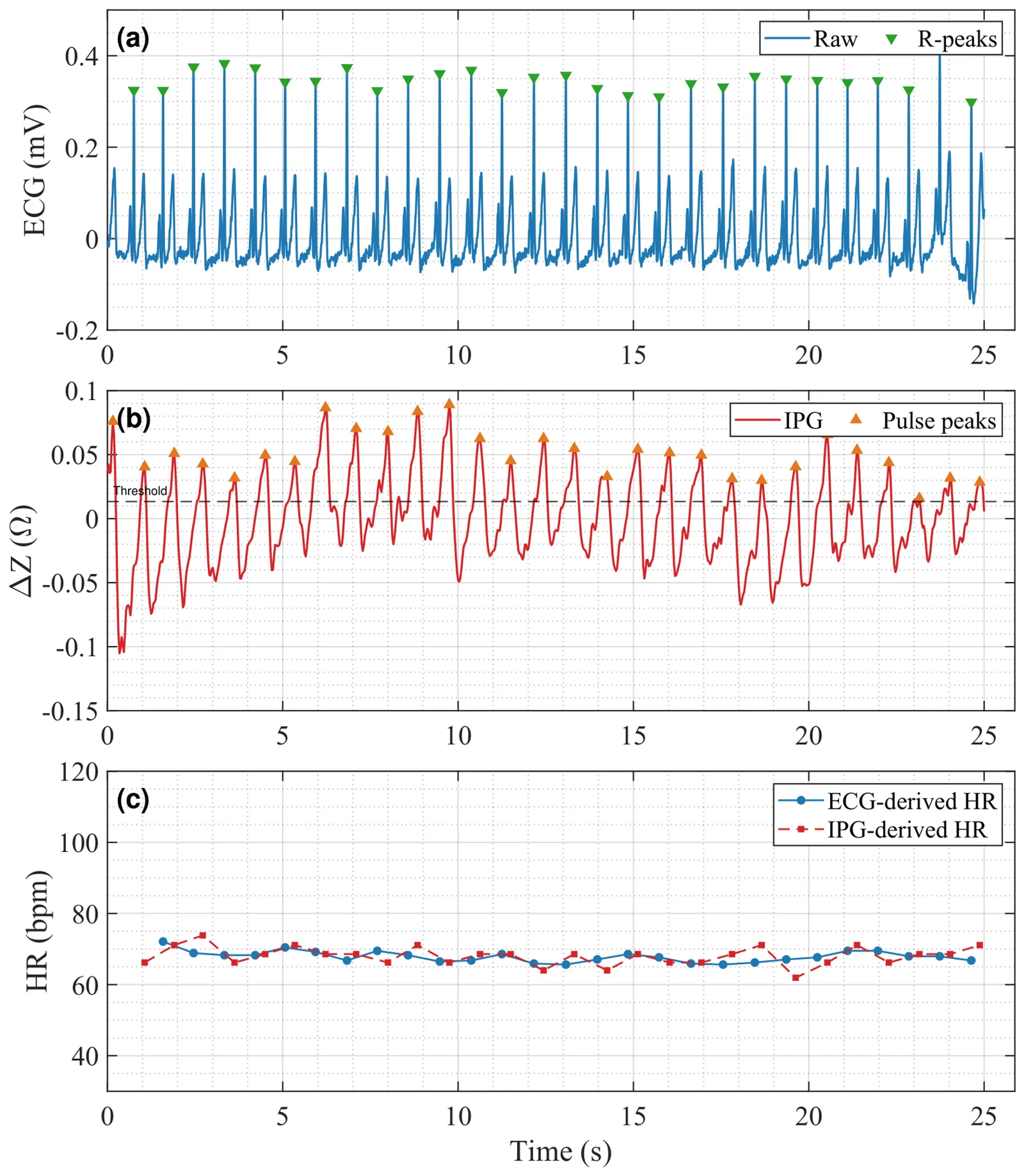
Results of heart-rate estimation. (**a**) Electrocardiogram (ECG) signal used as the reference. (**b**) Impedance plethysmography (IPG) signal measured by the WCIS. (**c**) Estimated heart rate from ECG and IPG signals.

Figure 8 presents a representative time-domain segment of approximately 25 s illustrating the simultaneous acquisition and processing of the ECG and IPG signals, along with the derived heart rate traces. Figure 8a shows the raw ECG waveform, which exhibits the characteristic morphology expected from a standard single-lead configuration, with clearly discernible R-peaks annotated directly on the signal. The amplitude range, spanning roughly −0.1 to +0.4 mV, is consistent with typical wrist-based electrode recordings, and the regular inter-peak intervals reflect a stable, resting cardiac rhythm throughout the recording window. Figure 8b displays the simultaneously acquired IPG signal, along with the threshold used for peak detection. The IPG waveform exhibits a quasi-periodic oscillatory pattern whose peaks align temporally with the ECG R-peaks, confirming that the impedance modulation captured by the interdigitated LIG electrodes is physiologically driven by the cardiac cycle. Notably, the IPG waveform exhibits a slightly lower signal-to-noise ratio than the ECG, as expected given the inherently smaller signal magnitude of impedance-based sensing at the wrist and the sensitivity of bioimpedance measurements to contact quality and motion artifacts. Nevertheless, the peak detection algorithm successfully identifies the cardiac-cycle-associated peaks with reasonable fidelity across the full recording segment.

Figure 8c overlays the beat-to-beat heart rate time series derived from both modalities. The ECG-derived HR serves as the reference, exhibiting values in the range of approximately 60–100 bpm with moderate temporal variability, reflecting natural heart rate fluctuations during the measurement. The IPG-derived HR tracks the ECG-derived reference reasonably well, capturing the general trend of the heart rate over time. Some degree of instantaneous discrepancy between the two traces is evident, possibly due to pulse-detection errors arising from noise or morphological variability in the IPG waveform. Nonetheless, the overall temporal variation between the two HR series provides qualitative evidence that the LIG-based IPG sensor can extract physiologically meaningful cardiac information from the wrist.

**Figure 9:**
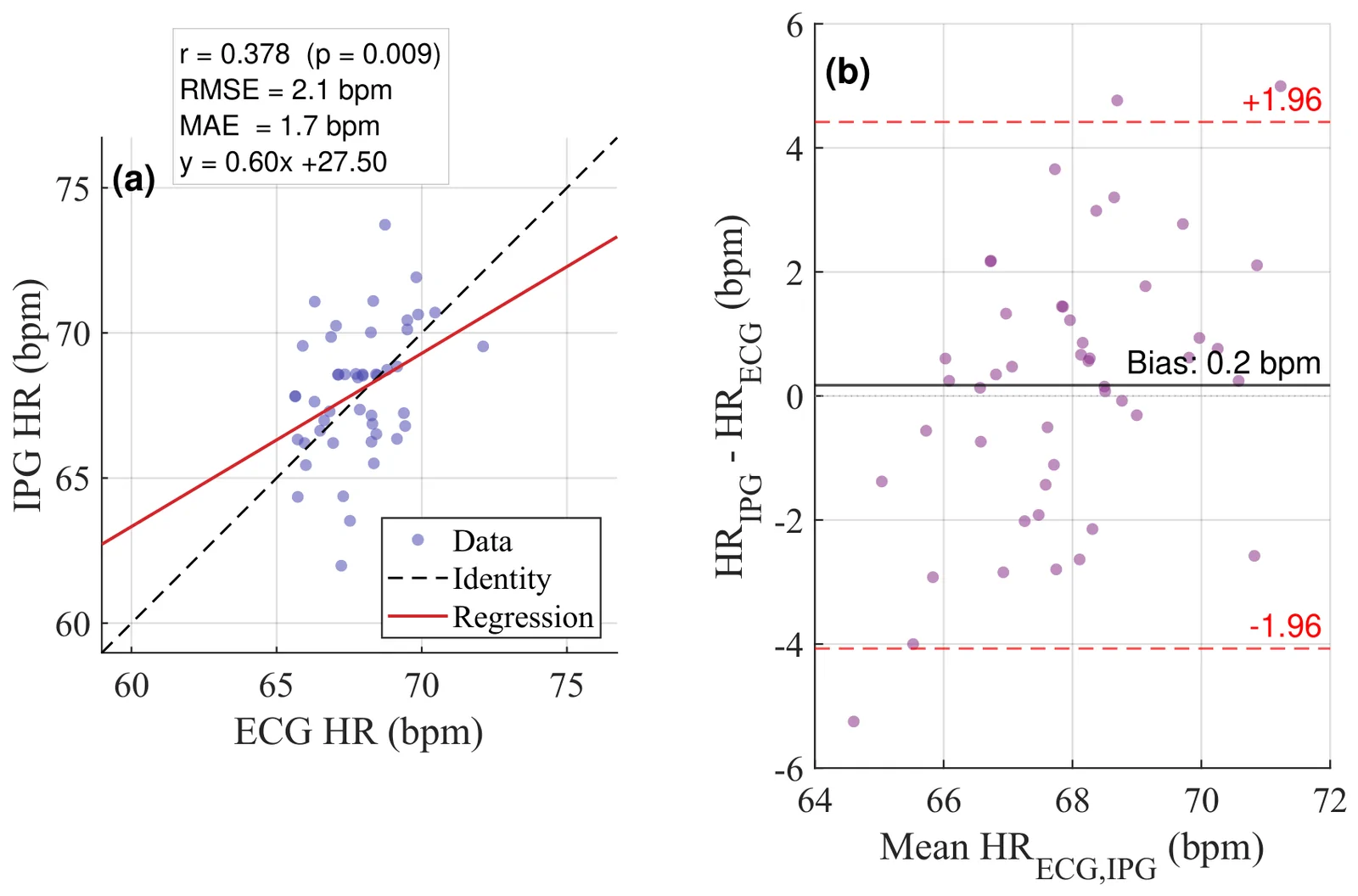
Statistical analysis of heart-rate estimation. (**a**) Correlation plot between IPG and ECG-derived heart rate. (**b**) Bland–Altman plot for inter-method comparison.

To go further, Figure 9 provides a quantitative statistical evaluation of the agreement between IPG-derived and ECG-derived heart rate estimates. Therein, Figure 9a shows a scatter plot of the IPG HR values as a function of the corresponding ECG HR values across all data points, with the identity line (dashed line) and the linear regression fit (continuous line). The data points are distributed around the identity line, though with visible scatter, which is numerically summarized by a root mean square error (RMSE) of 2.1 bpm and a mean absolute error (MAE) of 1.7 bpm. These metrics indicate that, on average, the IPG-derived HR deviates from the ECG reference by less than 2 bpm, which falls within clinically acceptable thresholds for resting HR monitoring in wearable devices. The Pearson correlation coefficient of r=0.378(p=0.009) indicates a statistically significant positive correlation between the two modalities. However, the moderate magnitude of r also reflects the sensor’s inherent limitations under the tested conditions, including the relatively narrow HR range covered (approximately 60–75 bpm), which naturally constrains the dynamic range available for establishing a strong linear correlation.

Figure 9b depicts a Bland–Altman plot, which offers a deeper assessment of inter-method agreement by examining the difference between IPG and ECG HR measurements as a function of the mean of the two modalities. The mean bias is 0.2 bpm, indicating that IPG introduces negligible estimation error with respect to the ECG reference. The limits of agreement (LoAs), defined as the mean bias ±1.96 standard deviations, span a range of approximately ±6 bpm, while this spread reflects random measurement variability, the remaining bias near zero indicates the absence of proportional error across the HR range examined. The relatively uniform distribution of differences across the mean HR axis suggests that the discrepancies are largely stochastic rather than systematically dependent on heart rate magnitude. This rationale is consistent with random noise and intermittent peak detection errors rather than with a structural limitation of the sensor per se.

**Figure 10:**
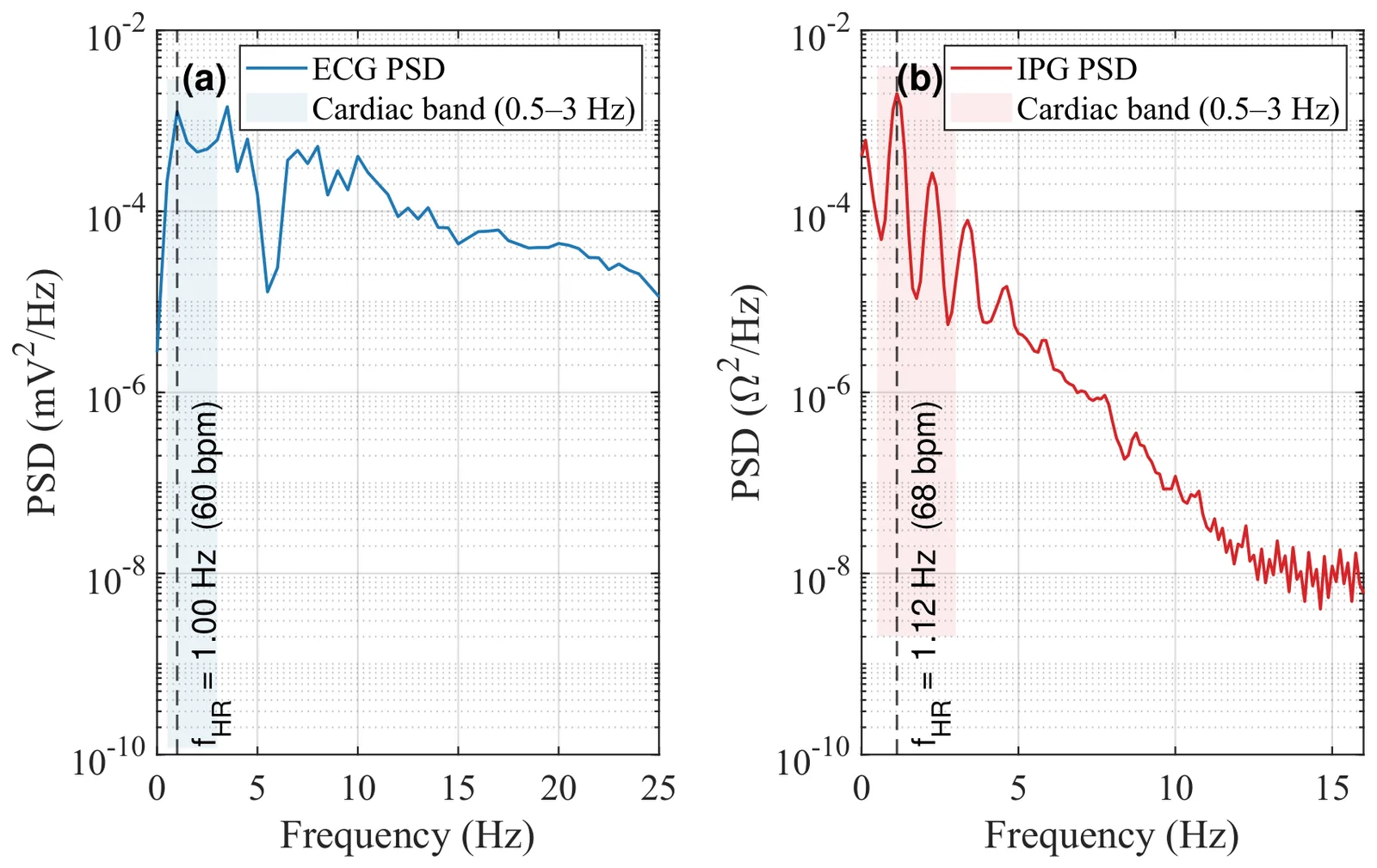
Spectral analysis for heart-rate estimation. (**a**) Spectrum of the ECG signal. (**b**) Spectrum of the IPG signal.

Figure 10 provides complementary frequency-domain evidence supporting the physiological validity of the IPG signal acquired with the LIG electrodes. Figure 10a shows the power spectral density (PSD) of the ECG signal on a logarithmic scale, computed over the full signal. A prominent spectral peak is observed at $f_{\mathrm{HR}}=1.00$ Hz, corresponding to a heart rate of 60 bpm, within the highlighted cardiac band spanning 0.5–3 Hz. The sharpness and prominence of this peak above the noise floor confirm the high signal quality of the ECG reference used for comparison. Figure 10b shows the PSD of the IPG signal, also on a logarithmic scale. Therein, a spectral peak is identifiable at $f_{\mathrm{HR}}=1.12$ Hz, corresponding to a heart rate of approximately 68 bpm. The small frequency offset between the ECG and IPG peaks is consistent with the known interbeat variability observed throughout the recording. Interestingly, the cardiac-band peak in the IPG-based PSD rises clearly above the background noise floor, confirming that the interdigitated LIG electrode configuration successfully transduces pulsatile hemodynamic activity into a measurable impedance signal at the wrist. The presence of this well-defined spectral component confirms the time-domain and statistical analyses, thus demonstrating that the LIG-based IPG sensor encodes reliable heart rate information despite the signal acquisition challenges inherent to wrist-based bioimpedance measurements.

## 4. Conclusions

This study demonstrates the viability of a wrist-worn cardiac impedance sensor (WCIS) based on laser-induced graphene (LIG) interdigitated electrodes as an effective solution for non-invasive cardiovascular monitoring. The methodology employed direct laser writing with a 405 nm laser on a polyimide substrate, optimizing the process over five scanning cycles to obtain a structure with a low series resistance of 88.703 Ω and homogeneous structural properties. Using impedance plethysmography (IPG), the device captured variations in blood volume with enough stability, achieving a signal-to-noise ratio (SNR) of 83.4 dB and a relative variability of only $6.8\times 10^{-3}$% in the absence of physiological modulation. Finally, the system’s precision was validated by estimating heart rate, yielding a mean absolute error (MAE) of 1.7 bpm and an RMSE of 2.1 bpm relative to the gold standard (ECG), confirming its potential as a low-cost, high-fidelity tool for integration into wearable and flexible medical devices.

## Figures and Tables

**Table 1:** MAX30001 BioZ channel configuration settings.

Parameter	Value
Analog HPF Cutoff	1 kHz
INA Power Mode	Low Noise
Channel Gain	10 V/V
Sample Rate	32 Samples/s
Digital LPF Cutoff	4 Hz
Digital HPF Cutoff	0.5 Hz
Current Generator Mode	Chopped w/o LPF
Current Generator Monitor	Disabled
Generator Frequency	4 FMSTR (128 kHz)
Magnitude	96 μA
External Resistor Bias Enable	Internal

**Table 2:** MAX30001 ECG channel configuration settings.

Parameter	Value
Channel Gain	20 V/V
Sample Rate	512 Samples/s
Digital LPF Cutoff	40.96 Hz
Digital HPF Cutoff	0.5 Hz
Fast Recovery Mode	Normal
Fast Recovery Threshold	63 × 2048 LSB

**Table 3:** Comparison of Raman shift values obtained with a 405 nm laser with those reported in the literature for a 450 nm laser.

Peak	Raman Shift Obtained [${\mathrm{cm}}^{-1}$]	Raman Shift Reported [${\mathrm{cm}}^{-1}$]	Percent Error [%]
D peak	1350.81±1.53	1350	0.06
G peak	1583.32±2.98	1580	0.21
2D peak	2681.41±2.59	2690	0.32

## Data Availability

Data are contained within the article. The original contributions presented in this study are included in the article. Further inquiries can be directed to the corresponding author.
